# Harnessing eDNA metabarcoding to investigate fish community composition and its seasonal changes in the Oslo fjord

**DOI:** 10.1038/s41598-024-60762-8

**Published:** 2024-05-02

**Authors:** Cintia Oliveira Carvalho, William Gromstad, Micah Dunthorn, Hans Erik Karlsen, Audun Schrøder-Nielsen, Jonathan Stuart Ready, Torbjørn Haugaasen, Grete Sørnes, Hugo de Boer, Quentin Mauvisseau

**Affiliations:** 1 Natural History Museum, University of Oslo, Oslo, Norway; 2 Group for Integrated Biological Investigation, Center for Advanced Studies of Biodiversity, Federal University of Pará, Belém, Brazil; 3 Marine Research Station Drøbak, University of Oslo, Oslo, Norway; 4 Faculty of Environmental Sciences and Natural Resource Management, Norwegian University of Life Sciences (NMBU), P.O. Box 5003, 1432 Aas, Norway

**Keywords:** Ichthyology, Molecular biology, Biodiversity, Conservation biology

## Abstract

In the face of global ecosystem changes driven by anthropogenic activities, effective biomonitoring strategies are crucial for mitigating impacts on vulnerable aquatic habitats. Time series analysis underscores a great significance in understanding the dynamic nature of marine ecosystems, especially amidst climate change disrupting established seasonal patterns. Focusing on Norway's Oslo fjord, our research utilises eDNA-based monitoring for temporal analysis of aquatic biodiversity during a one year period, with bi-monthly sampling along a transect. To increase the robustness of the study, a taxonomic assignment comparing BLAST+ and SINTAX approaches was done. Utilising MiFish and Elas02 primer sets, our study detected 63 unique fish species, including several commercially important species. Our findings reveal a substantial increase in read abundance during specific migratory cycles, highlighting the efficacy of eDNA metabarcoding for fish composition characterization. Seasonal dynamics for certain species exhibit clear patterns, emphasising the method's utility in unravelling ecological complexities. eDNA metabarcoding emerges as a cost-effective tool with considerable potential for fish community monitoring for conservation purposes in dynamic marine environments like the Oslo fjord, contributing valuable insights for informed management strategies.

## Introduction

Ecosystems worldwide are changing at unprecedented rates, driven by anthropogenic activities including habitat degradation and modification, and/or climate change^1^. These changes significantly affect every ecosystem and are currently leading to a global biodiversity crisis, often described as the sixth mass extinction^2,3^. Aquatic habitats are among the ecosystems most vulnerable to these shifts, where there are many studies showing a decline in ecosystem functioning and alterations in species richness^4–7^. Marine environments in particular are being affected by the warming of ocean temperature, overfishing, and pollution^8–10^. Rising ocean temperatures and changes in seasonal temperature patterns can disturb the balance between predators and prey, impacting their survival^8^. Indeed, seasonal variations in temperature, nutrient availability, and daylight hours can significantly impact marine life, influencing migration patterns, reproductive cycles, and biodiversity^11,12^. Overall, marine fish abundance has declined by 38% since 1970^13^, and coastal ecosystems like seagrass and mangrove areas have diminished by over two-thirds^14^.

Monitoring seasonality and changes over time in marine environments is crucial for understanding the dynamic nature of these ecosystems^11^. In that regard, time series data collected over extended periods become especially valuable in unravelling relationships between communities and their environment^12,15^. In marine environments, where seasonal changes are pronounced, time series analysis helps identify recurring patterns and trends^16^. It allows the tracking of marine species migrations, phytoplankton growth, and variations in ocean currents^17,18^. Furthermore, the application of time series analysis can provide insights into trophic interactions. Such an example is the tracking variations in the interactions between California sea lions (Otariidae), Pacific jack mackerel (Carangidae), and their prey across seasons^19^. California sea lions are apex predators known to exhibit seasonal variations in their foraging behaviours, while Pacific jack mackerel serves as their prey species^19^. Such information is vital for predicting and managing the impacts of climate change that can disrupt established seasonal patterns^20^.

Traditional assessments of marine biodiversity have been applied for many years and often concentrate on macro-organisms and involve visual and manual methodologies, such as diver surveys, trawling, acoustic techniques, or aerial imagery^21^. However, this method can face limitations caused by its labour-intensive nature, as they are more time-consuming in the field, potentially disruptive, sometimes impactful in terms of mortality to marine species, and can miss rare or elusive species present in the area^15,22,23^. Recognizing these limitations, environmental DNA (eDNA) has emerged as a powerful tool to enhance our ability to conduct comprehensive time series analysis in marine ecosystems^12,24–26^. This now widely accepted method identifies the biota in a region by extracting, amplifying, and sequencing the DNA left by them in the environment (for example by filtering the DNA from a volume of water), allowing standardised detection of various organisms, thus providing a holistic picture of biodiversity changes over time^19,25,27^. This approach is particularly valuable in monitoring rare or elusive species that may not be easily observed through traditional methods^28,29^. Despite its growing popularity, eDNA technology still faces some challenges, including methodological complexities such as primer biases and incompleteness of reference databases, which need rigorous controls, and should be complemented where possible with external validation to develop standardised protocols^30,31^. Combining time series analysis with eDNA technology therefore offers a powerful approach to monitor seasonality and long-term changes in marine environments, contributing to more effective conservation and management strategies in the face of ongoing environmental challenges^15,19^.

Fjord ecosystems, particularly present in Norway, are dynamic environments characterised by their unique geographical features, contributing significantly to global biodiversity^32^. Fjords are deep, glacially-carved inlets that form a transitional zone between fully marine and freshwater ecosystems^33^, and Norwegian fjord in particular supports a wide array of marine and freshwater species^34^. The biodiversity in these ecosystems is particularly rich due to the presence of various niches and habitats along the fjord gradients^35^. From the deep, saline waters near the fjord mouths to the less saline and often brackish waters further inland, fjords encompass a spectrum of conditions that foster a diverse range of flora and fauna^36^. This interface between marine and freshwater ecosystems within fjords contributes to the remarkable biodiversity seen in Norway's fjordic landscapes, making them prime areas for ecological research and conservation efforts^35,36^. The Oslo fjord in southern Norway has endured significant historical impacts over the past century, as technological advancements in fisheries have exerted considerable pressure on the local fish community, leading to a dramatic reduction in fish abundance within the fjord^37–40^. Other environmental challenges include eutrophication as a result of various pollution, mainly caused by untreated sewage, industrial waste, and agricultural runoff. These excessive nutrient inputs have led to harmful algal blooms and oxygen depletion^40,41^. In addition, climate change is also affecting the fjord, causing rising temperatures and sea levels, and changes in precipitation patterns^39,42^. Given the complexity of the challenges facing the Oslo fjord, accurate monitoring of its resident species is important.

In this study, we used molecular-based detection methods, to investigate the aquatic fish community and their seasonal changes within the Oslo fjord during a one year period, with bi-monthly sampling along a transect. Additionally, we made a comparison between the SINTAX and BLAST+ approaches for taxonomic assignment, to evaluate if any significant differences are found between the two methods. Our findings contribute to the broader body of knowledge essential for informed conservation and management strategies, with direct implications for the sustainability of the Oslo fjord and similar aquatic environments facing analogous challenges.

## Methods

### Study site

**Figure 1:**
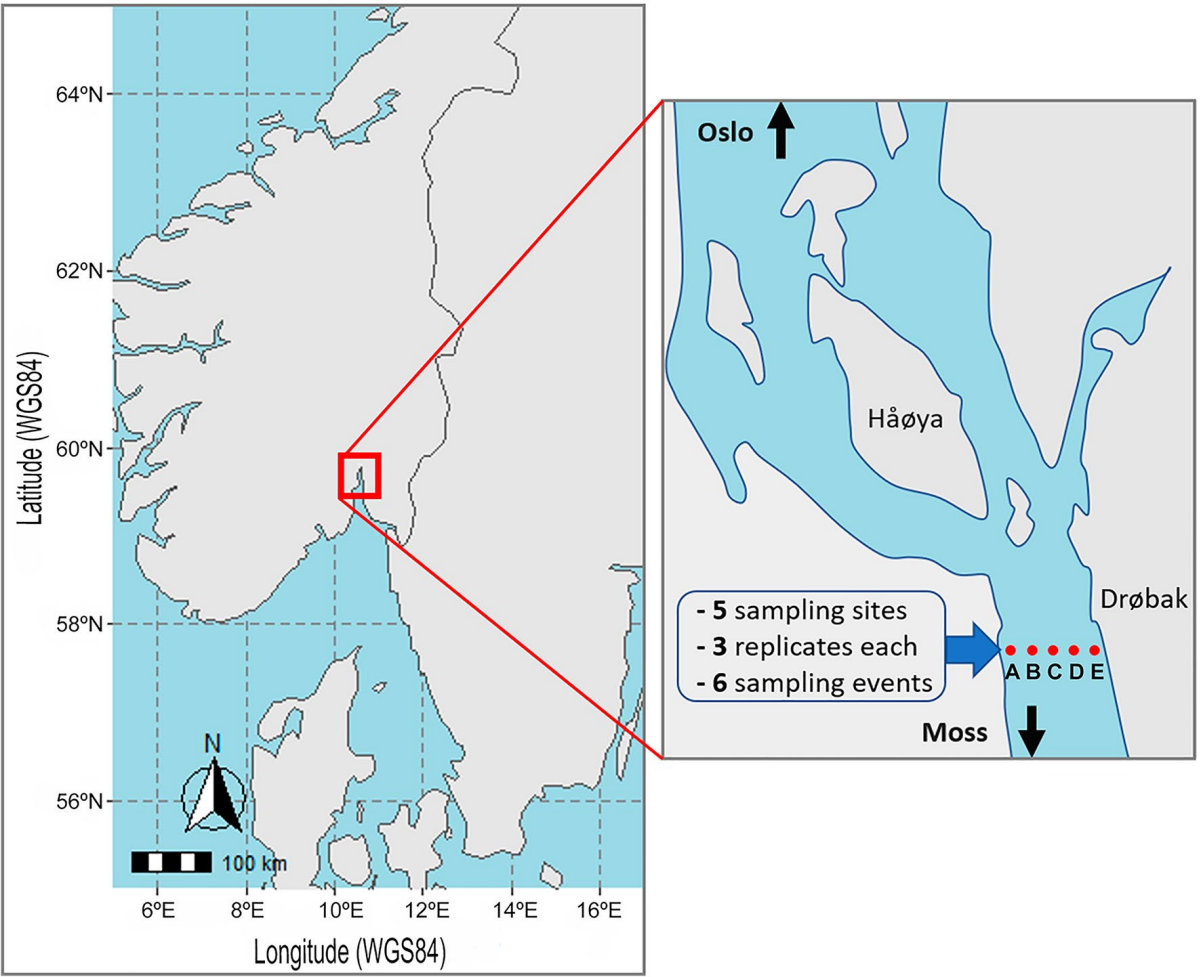
Sampling sites across the Oslo fjord. Sampling sites are indicated with red dots crossing the fjord outside Drøbak. The width of the fjord bottleneck near Drøbak is approximately 1.5 km. The first part of the map was created using R ver. 4.3.3 (https://cran.r-project.org/) with the *ggplot2* and *sf* packages, and the other part is a drawing made through PowerPoint to show the sampling location.

eDNA sampling was performed every two months from March 2022 to January 2023 (Supplementary Table S1), resulting in six sampling events, along a transect encompassing five sampling locations, spaced 200 m apart, across the strait near the city of Drøbak in the Oslo Fjord (Fig. 1). Oslo fjord, stretches 100 kms from Skagerrak and the North Sea to Oslo and reaches a maximum depth of 258 m. The inner fjord is separated from the outer fjord by the strait near Drøbak which has a shallow sill (20 m) that severely limits the exchange of deep water between the fjord basins^43,44^.

### eDNA sampling

At each sampling location, 15 L of surface water was collected in sterile jerry cans from a boat, and taken back to the Marine Research Station in Drøbak for processing. There, water from each location was filtered through three independent 0.8 μm Whatman (Cytiva, USA) Mixed Cellulose Ester filters (22 mm diameter) using sterile 22 mm Swinnex (Merck Millipore, Germany) Filter Holders and a Vampire Sampler (Bürkle GmbH, Germany) pump system. The volume of water filtered for each filter ranged from 1000 to 2000 ml and can be found in Supplementary Table S1, along with the data for the measured environmental variables: temperature °C, conductivity mS, salinity, total dissolved solids (TDS) g/L, and pH, collected at each site. For negative controls, one litre of distilled water was filtered at each sampling event at the field station. Following water filtration, each filter was air dried and stored in a 2 ml DNase and RNase-free tube at − 20 °C until DNA extraction. Sterile equipment and disposable gloves were used when processing water collected at each location to avoid potential cross-contamination between sites.

### eDNA metabarcoding analysis

A total of 96 unique eDNA samples (i.e. 90 eDNA samples and 6 negative field controls) were collected (see Supplementary Table S1). DNA extractions were completed in a dedicated PCR-free room using the QIAGEN Blood and Tissue Kit (Qiagen, Netherlands) following the manufacturer’s instructions with slight modifications (i.e. volumes of ATL buffer, proteinase K, AL buffer and absolute ethanol were doubled to ensure an efficient extraction of all eDNA samples and the lysis step was overnight). DNA amplification of short regions of the mitochondrial 12S rRNA was conducted using the following metabarcoding primers: a 172 bp fragment was targeted using the MiFish‐U‐F 5′‐GCCGGTAAAACTCGTGCCAGC‐3′ and MiFish‐U‐R 5′‐CATAGTGGGGTATCTAATCCCAGTTTG‐3′ primers^45,46^ (later referred as MiFish primers); and an approximately 182 bp fragment (from 170 to 185 bp) was targeted using the Elas02F/R 5′-GTTGGTHAATCTCGTGCCAGC-3′/5′-CATAGTAGGGTATCTAATCCTAGTTTG-3′ primer pair^47^ (later referred as Elas02 primers). The selection of these primers was based on their applicability and resolution for assessing fish communities (including both ray finned fishes and elasmobranchs) as well as other aquatic vertebrates potentially present in Oslo fjord^47–51^.

PCR amplifications were conducted in triplicate, and additional negative controls, also called NTCs (Non Template Controls, i.e. samples where the template DNA was replaced with ddH2O at the PCR step) were included to ensure the absence of contamination. PCRs were conducted with already indexed primers following Fadrosh et al. 2014^52^. PCR conditions were as follows: 10 μl of 2X Accustart Toughmix II (QuantaBio, USA), 0,5 μl of each indexed primer (10 μM each), 7 μl of nuclease free water and 2 μl of extracted eDNA in a final volume of 20 μl. The PCR amplification parameters were as follows: initial denaturation at 94 °C for 3 min, then 35 cycles of 94 °C for 20 s, 52 °C for 20 s and 72 °C for 30 s and then the final extension of 72 °C for 3 min. PCR products were visualised on 1% agarose gels and quantified using ImageLab Software v6.0 (Bio-Rad Laboratories, USA)^53^. To ensure equal representation of all amplicons, uniform amounts of each amplicon were merged using a Biomek4000 liquid handling robot (Beckman Coulter, USA). The DNA amplicon pool was then cleaned using 10% of Illustra ExoProStar (Cytiva, USA) and 1.0X AMpure XP (Beckman Coulter, USA) bead clean before performing size selection using BluePippin (Sage Science, USA) targeting the respective marker size. A final quality control was done by visualising the library on a Fragment Analyzer (Agilent, USA) using the High Sensitivity Genomic DNA Kit before the prepared library was sequenced on a Miseq platform (Illumina, USA) using a 250 bp paired-end reads kit.

### Bioinformatics and data analysis

Bioinformatics processing and filtering (i.e. merging, demultiplexing, and data cleaning steps) was conducted as in^53^, with slight modification regarding denoising and the taxonomic assignment steps. Here, the unique sequences were clustered at 97% similarity threshold using USEARCH v11.0^54^ to form OTUs (Operational Taxonomic Units). Despite the acknowledged risk of signal loss at this similarity percentage, we opted for this threshold due to its established effectiveness and prevalence in the field^20,55,56^. Subsequently, the taxonomy assignment was done using the k-mer based approach SINTAX in VSEARCH v2.21.1 ^57,58^, where the clustered OTUs were assigned with a minimum 90% similarity threshold. Additionally, aiming to see if there is a difference in taxonomic assignment results, we also performed the taxonomic assignment step for all OTUs using the alignment based approach BLAST+ v2.14.1^58,59^, using the same 90% minimum similarity threshold. While recognizing that a 90% similarity match does not guarantee species-level assignment^60^, the preliminary output dataset predominantly exhibited percentage similarities exceeding 96% at the species level, bolstering confidence in our taxonomic assignments for subsequent analyses. Any matches falling below this threshold were excluded from further consideration, which resulted in the absence of non-species level assignments in the final dataset for the area. European waters, including the Oslo Fjord, have one of the most complete reference databases considering the biodiversity that is known for the region, enhancing the reliability of our assignment results^61^. For sequences with multiple hits, we conducted manual verification of species assignments through NCBI Blast searches and consulted relevant literature for the study area to ensure accuracy.

The SINTAX approach was chosen here, as it operates by utilising random samples of short DNA base combinations to assign taxonomic labels to query sequences^54^. Unlike some other methods, SINTAX requires an exact match between these short DNA sequences and reference databases for accurate taxonomic assignments^54,58^. This approach is particularly suitable for sequences with long conserved regions, such as ribosomal genes, where the presence of distinct patterns can aid in precise classification^54,58^. On the other hand, the BLAST+ algorithm employs an overall alignment score-based method for taxonomic assignment^58,59^. Instead of relying solely on exact matches, BLAST+ compares query sequences against a reference database using a heuristic algorithm^59^. This algorithm identifies regions of similarity between the query and database sequences, allowing for more flexible matching, even in cases where there isn't an exact match^58,59^. Both taxonomic assignments were done based on the MIDORI2 database (MIDORI_UNIQUE_NUC_GB257_srRNA_SINTAX_20230814), as this new database offers highly reliable taxonomic identification of eDNA reads by having the sequences extracted from the GenBank BLAST NT database^62^ with inaccurate annotations removed^58^. Employing the SINTAX and the BLAST+ approaches allowed a more comprehensive understanding of taxonomic assignment outputs, ensuring reliable taxonomic identifications in our eDNA analysis.

In order to remove potential false positives, contaminants, or sequencing errors, we filtered the data removing the maximum number of reads detected in negative controls for each associated OTU across all samples^63^. Additionally, replicates from each sample, and subsequently, the replicates from each sampling site were combined. Species that were not the primary focus of our study were excluded, as well as OTUs that were likely a result of carry-over contaminations^63,64^. Our analysis specifically concentrated on retaining OTUs associated with fishes (including both ray-finned fishes and elasmobranchs), whales, and dolphins. Statistical analyses and results visualisation were made using RStudio v4.2.3^65^. The relative read abundance (RRA) was obtained for each OTU by dividing the number of OTUs copies by the sum of the number of copies of all OTUs in the sample^66–68^. Plots for each marker were generated using *ggplot2* v3.4.3^69^. The heatmap was created with the presence/absence data matrix to visualise the frequency of occurrence of species using the *heatmap3* v1.3.7, *RColorBrewer* v1.1.2, and *Phyloseq* v1.42.0 packages^70^. To see if increasing the number of field replicates or PCR replicates would increase the number of detected species, rarefaction curves were created with 1000 bootstraps in iNEXT Online^71^. ANOVA was used to assess differences in species richness detected by the different taxonomic assignment approaches, and a Q-Q plot and a Levene’s test were done prior to the analysis to check if the data was normally distributed with equal variances. Permutational multivariate analysis of variance (PERMANOVA) was performed based on Bray–Curtis similarity, using the *adonis2* function from the *vegan* package v2.6.4^72^ in R, to test patterns of dissimilarities among fish species composition depending on sites and sampling events, and data was visualised in boxplots. We then employed a generalised linear model (GLM) to investigate the relationship between species richness and the volume of water filtered, temperature, conductivity, salinity, total dissolved solids (TDS), and pH (see Supplementary Table S1), and also between species composition and sampling events. For key species expected to frequent the Oslo fjord, seasonal patterns were visualised in a Bubble plot generated in *ggplot2* v3.4.3^69^, considering the combined DNA reads from Elas02 and MiFish datasets.

## Results

A total of 14,314,724 raw reads were obtained following metabarcoding amplification and sequencing, where 6,364,113 reads were assigned to Elas02 and 5,153,578 to MiFish primers. After quality filtering, considering all taxa detected, 4,714,351 reads remained for Elas02 and 4,811,060 reads for MiFish, where around 50% of the reads were from Humans. Retaining only the OTUs from fishes (both ray finned fishes and elasmobranchs), whales, and dolphins for our analysis, a total of 325,252 reads were assigned to the Elas02 primer set and 100,172 reads were assigned to the MiFish primer set. Taxonomic assignment using SINTAX and combining the results from the two primer sets resulted in the identification of 36 families, 59 genera, and 63 unique fish species, as well as the sei whale (*Balaenoptera borealis*) and harbour porpoise (*Phocoena phocoena*). Of the 65 species, 35 were detected by both primers, while only 16 and 14 species were detected by Elas02 and MiFish respectively (see Supplementary Fig. S1). Both datasets additionally detected sequences originating from diverse organisms, including birds and mammals (see Supplementary Table S2).

**Figure 2:**
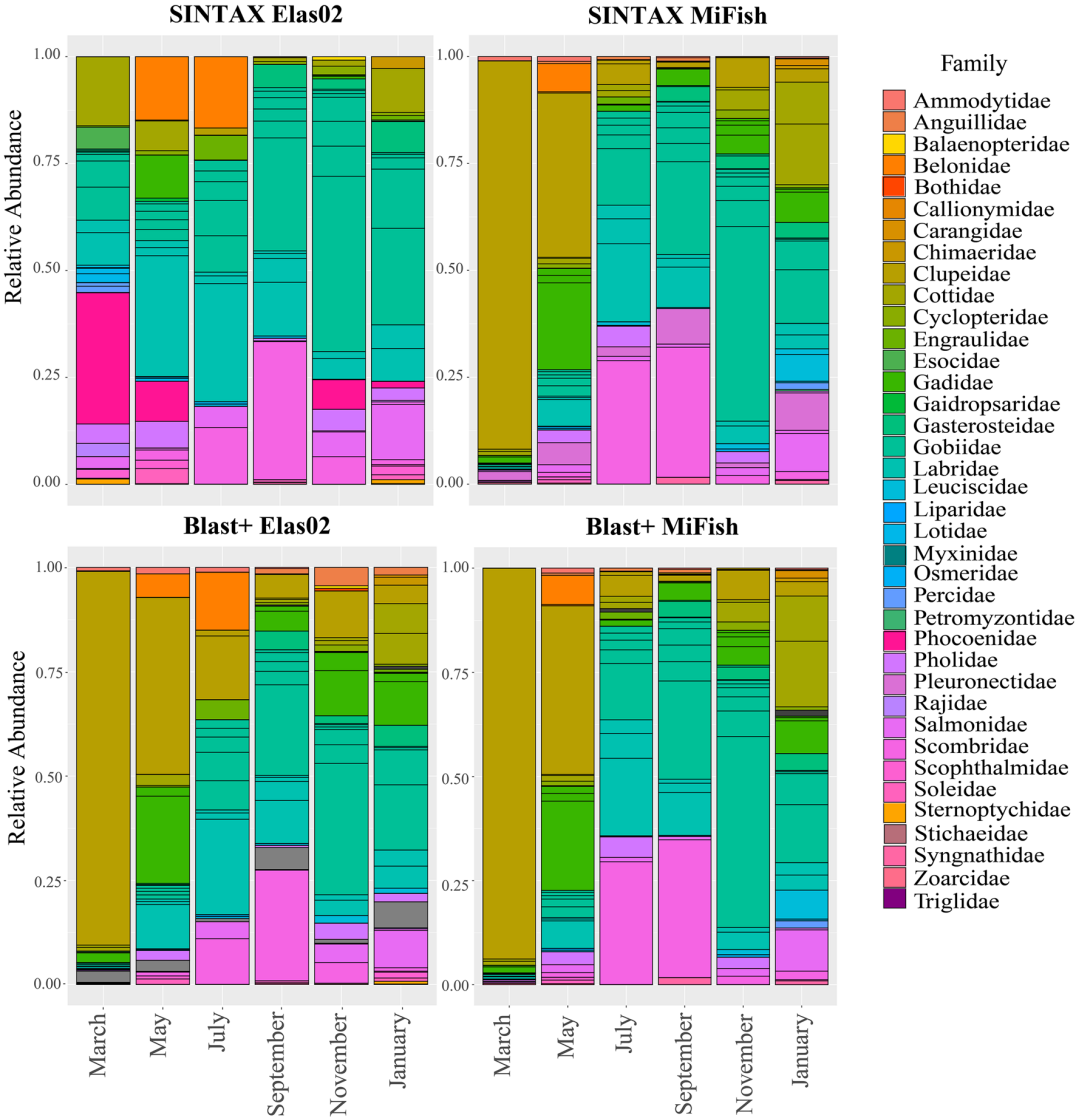
Relative read abundance of fish at family-level resolution detected through eDNA metabarcoding, at each seasonal sampling event for each marker, and for each taxonomic assignment method.

Variability in species composition was observed across the different sampling events, and between the Elas02 and MiFish primer datasets (see Supplementary Fig. S1 and Fig. 2). No statistical differences were found in the species detected between the SINTAX and BLAST+ approaches (ANOVA, F = 0.008, *p* = 0.927). Considering this, we used the SINTAX assignment for subsequent analyses, as it is indicated as the best method for 12S (Leray et al., 2022). Using the SINTAX algorithm, the families Belonidae, Cottidae, Gobiidae, Phocoenidae, and Scombridae presented the highest read abundances for Elas02, in contrast, the families Clupeidae, Gadidae, Scombridae, Gobiidae, and Labridae had the most abundant read counts for MiFish. In the March sampling event, Elas02 reads were dominated by the harbour porpoise (Phocoenidae), whereas MiFish reads were dominated by representatives of the herring/sardine family Clupeidae (Fig. 2). In the Blast+ approach, a similar result was found, with only one more family identified, Stichaeidae (Fig. 2, see Supplementary Table S3). Clupeidae was the family with the most abundance of reads for both markers, which is a different result than that obtained using the SINTAX assignment for Elas02. Most reads for the family were found in samples from the March and May collection events (Fig. 2). Rarefaction curves reached plateaus at about 25 repeated samples for the field replicates and at ~ 50 repeated samples for the PCR replicates, indicating adequate recovery of the total diversity (Supplementary Fig. 2).

**Figure 3:**
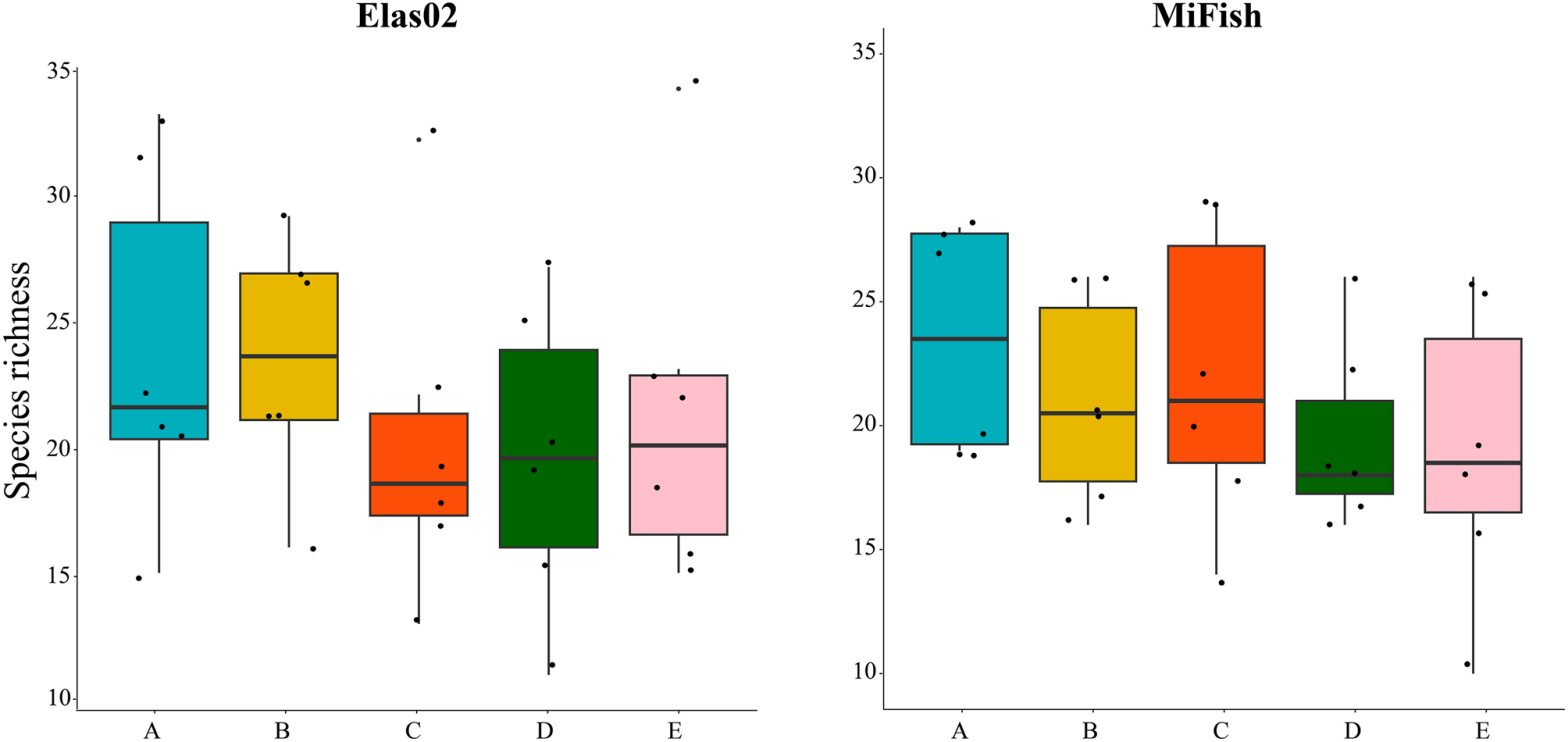
Boxplots showing the mean, quartiles, and 95% confidence limits for the number of species identified at the different sites, based on the totals across all six seasonal sampling events.

When considering species richness between the different sites, no significant difference was found for both Elas02 (PERMANOVA, R^2^ = 0.084, *p* = 0.724) and MiFish (PERMANOVA, R^2^ = 0.113; *p* = 0.549) (Fig. 3). Species detection was significantly affected by the volume of filtered water (Elas02, β = 0.012, *p* < 0.01; MiFish, β = 0.009, *p* < 0.05), the temperature (Elas02, β = − 7.292, *p* < 0.001; MiFish, β = − 6.763, *p* < 0.001), and the conductivity (Elas02, β = 10.895, *p* < 0.01; MiFish, β = 10.026, *p* < 0.001) of the water at each sampling event.

**Figure 4:**
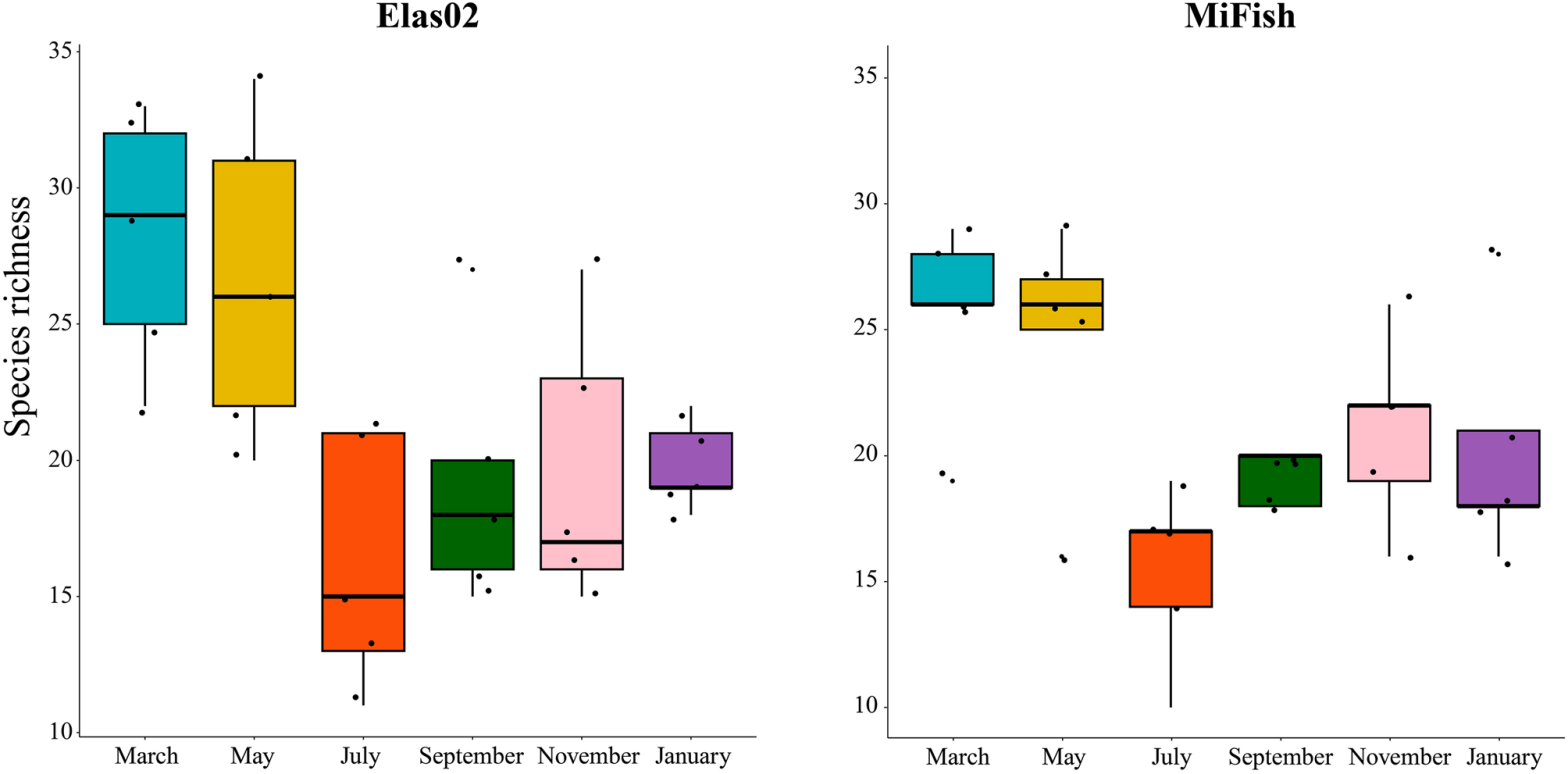
Boxplots showing the mean, quartiles, and 95% confidence limits for the number of species identified at each sampling event and within Elas02 and MiFish markers.

There was strong evidence for seasonal variation in species richness for both datasets (PERMANOVA Elas02, R^2^ = 0.492, *p* < 0.01; MiFish, R^2^ = 0.470, *p* < 0.01) (Fig. 4). When considering which months had significantly different species richness, the GLM indicated some differences in alpha diversity during the seasonal sampling (Fig. 4). For Elas02 there was very strong evidence for low species alpha diversity in January (β = 19.800, *p* < 0.001), strong evidence for high alpha diversity in March (β = 8.400, *p* < 0.01), and moderate evidence for high alpha diversity in May (β = 6.800, *p* < 0.05). For MiFish there was very strong evidence for low species alpha diversity in July (β = −10.200, *p* < 0.001), moderate evidence for low species alpha diversity in September (β = −6.400, *p* < 0.05) and January (β = −5.400, *p* < 0.05), and strong evidence for high alpha diversity in March (β = 25.600, *p* < 0.001).

**Figure 5:**
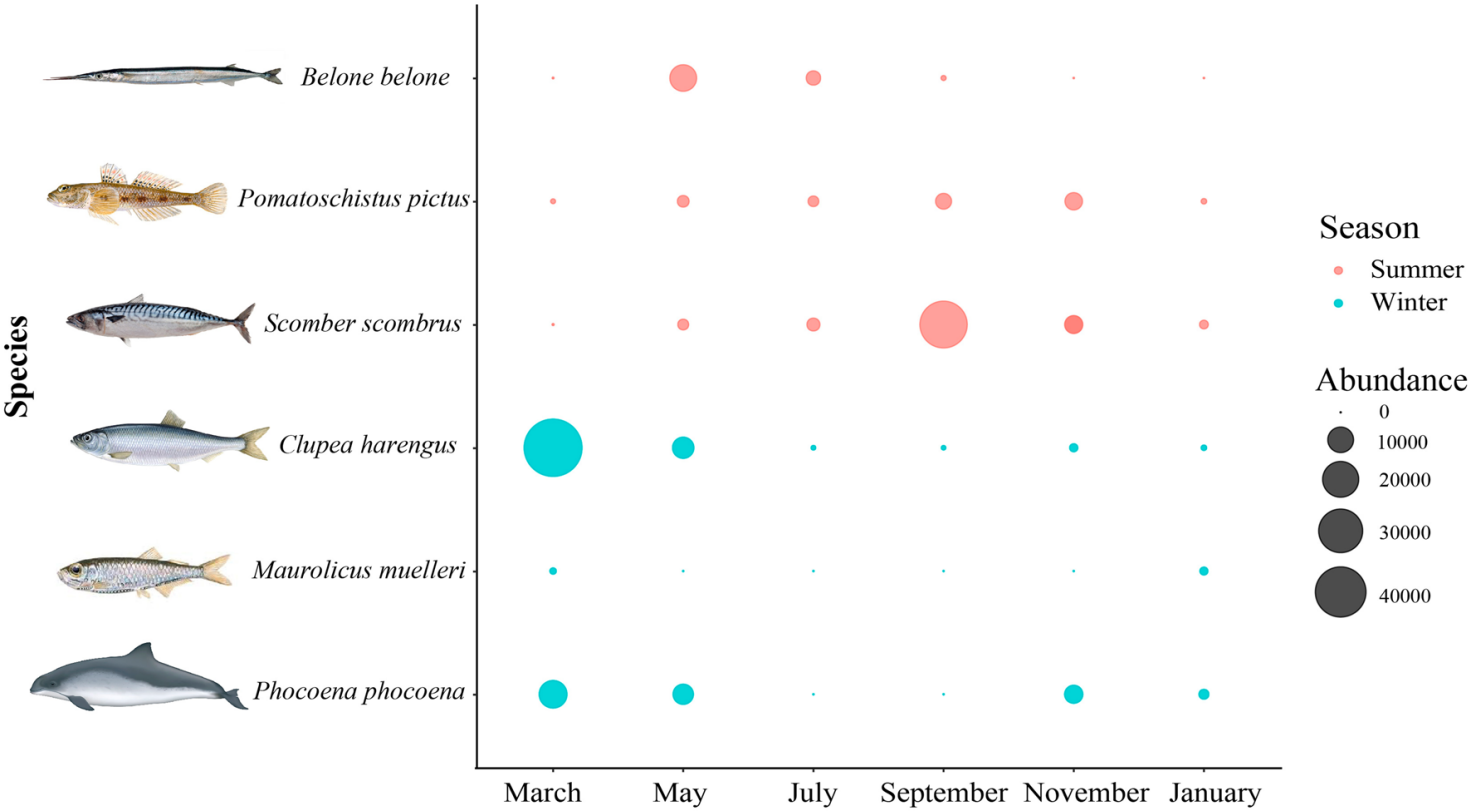
Seasonal changes in DNA read counts identified for expected species in the samples. Red bubbles indicate species mostly present in the Oslo fjord during the summer, while blue bubbles show species present during winter.

Considering the Elas02 and MiFish datasets combined, three species were mainly found during the summer sampling events (May, July, and September) (Fig. 5, see Supplementary Table S4), including garfish (*Belone belone*), Atlantic mackerel (*Scomber scombrus*), and painted goby (*Pomatoschistus pictus*). Reads assigned to the painted goby were found in relatively high numbers (over 1200 reads) in each sampling event, but samples from March and January had less than 300 reads (Fig. 5, see Supplementary Table S4). Large numbers of reads of eDNA assigned to the harbour porpoise (*Phocoena phocoena*) were found in March, May, November, and January (9982 reads, 5202, 4119, 1084, respectively), but no reads were assigned to this species during the warmest months of July and September. Reads assigned to Mueller’s pearlside (*Maurolicus muelleri*) were almost only identified in samples from March and January (398, and 668 reads), with the exception of a single read in July. Reads assigned to the Atlantic herring (*Clupea harengus*) were present in all samples throughout the year, but the number of reads varied a lot (see Supplementary Table S4): 44,871 and 5756 reads were found in March and May, but in the remaining months less than 700 reads were identified for this species in each seasonal collection event (see Supplementary Table S4).

## Discussion

This is the first temporal study using eDNA-based monitoring in the Oslo fjord with the aim of optimising and improving the standardisation of biodiversity monitoring in fjord ecosystems. We were able to successfully characterise the fish community present in the Oslo fjord, and simultaneously recovered seasonal changes in the dominant species identified (based on observed changes in read counts) that coincide with the known migratory cycles and habitat use of some marine species. This underscores the value of eDNA metabarcoding for biodiversity characterization and ecosystem monitoring. The taxonomic assignment of 12S ribosomal subunit RNA gene fragments performed using both the k-mer based approach SINTAX and the alignment based BLAST+ approach revealed no significant differences (ANOVA, F = 0.008, *p* = 0.927) in species richness. Only the family Stichaeidae was additionally identified with BLAST+. This finding could be related to the more conservative approach of SINTAX that requires an exact match between the query sequence and one or several sequences of the database^58^, leading to the lack of recognition of these families. The decision to include the SINTAX taxonomic assignment method adds rigour to our analysis, as the SINTAX algorithm identifies the top hit in a reference database, increasing the reliability for taxonomic prediction^54,58^.

As well as the detection of 63 unique fish species, the combined use of Elas02 and MiFish primer sets also unveiled the presence of iconic marine mammals, the endangered sei whale (*Balaenoptera borealis*) and the harbour porpoise (*Phocoena phocoena*) (see Supplementary Fig. S1). The sei whale is renowned for its migratory journeys, occupies a substantial role in trophic levels in marine food webs, and was recently listed as a threatened species^73^. Similarly, the harbour porpoise, a small cetacean with a coastal distribution, is often the focus of conservation efforts due to its susceptibility to anthropogenic threats^74^. The identification of these species underscores the potential of eDNA approaches to capture not only fish biodiversity but also the broader marine fauna^75^. The identification of 35 species shared between both primers showcases their combined effectiveness, yet the unique species detected by Elas02 (16) and MiFish (14) underscores the complementary nature of these primer sets. This aligns with current literature emphasising the importance of employing multiple primer sets for a more comprehensive assessment of biodiversity in environmental samples^49,51,76–79^.

Notably, our combined dataset played a crucial role in identifying elasmobranchs, including the vulnerable *Amblyraja radiata* (thorny skate)^80^. As elasmobranchs are susceptible to environmental changes and anthropogenic threats, their detection through our eDNA efforts provides valuable conservation insights^81^. Among the Chondrichthyes identified, the vulnerable rabbitfish or ratfish *Chimaera monstrosa*^82^ stands out. It exhibits a bathydemersal to benthopelagic distribution, seasonal migrations from deeper southern to inshore northern waters, primarily feeding on bottom-living invertebrates, and is commonly caught as by-catch in shrimp trawling in the North Sea and Skagerrak, highlighting its ecological relevance to the broader understanding of the ecosystem's biodiversity^83,84^. In addition to these unique species, expected important species for the Oslo fjord were also detected including the commercially important Atlantic cod (*Gadus morhua*) which is a key link in the North Atlantic food web^85^ along with the Atlantic herring (*Clupea harengus*) and the Atlantic mackerel (*Scomber scombrus*). Furthermore, the gobies *Pomatoschistus microps*, *Pomatoschistus minutus*, and *Pomatoschistus pictus*, indicate a diverse community of fish. Overall, the list of species identified represent different ecological niches and trophic levels, indicating that the method has likely captured the overall functional biodiversity and ecological complexity of the Oslo fjord.

The rarefaction curves of our individual sampling replicates and PCR reactions indicated that sequencing depth was sufficient to recover the species occurrence plateau. This aligns with the importance of comprehensive sampling efforts to accurately reflect ecosystem diversity^86^. Regarding the species dynamics in the Oslo fjord, we detected no significant variations in species read abundance across the sites. On the other hand, our data have shown that the volume of filtered water, temperature, and conductivity can influence the detection of fish species in marine environments. The influence of water temperature aligns with other studies indicating higher eDNA detection rates at elevated temperatures^87–89^. Additionally, it is worth considering the potential impact of dilution and inhibition effects resulting from increased freshwater inputs during the summer months; these factors may influence the detectability of fish species in the marine environment, particularly in areas where freshwater and seawater meet^36^. The observed temporal patterns in species composition, especially during the March sampling event, align with broader ecological studies emphasising the impact of seasonality on community dynamics^27,90^. Species in the family Clupeidae were highly abundant in the March and May sampling events. This family, represented here by the Atlantic herring (*Clupea harengus*) and the European pilchard (*Sardina pilchardus*), were particularly informative considering the dominance of herring, which contributed most of the eDNA that was identified. The observed peak abundance of Atlantic herring in March, coincides with its spawning period, underscoring the potential for enhanced eDNA signal strength during this time, as the increased liberation of herring sperm contributes to a more concentrated and detectable genetic footprint in the water^91–93^. The relative abundance of clupeids (specifically herring) and mammals varied considerably between the MiFish and Elas02 datasets, with MiFish generally amplifying more fish DNA than mammal DNA, while Elas02 detected greater amounts of mammal (mainly harbour porpoise) DNA, apparently impacting the relative abundance of herring (Supplementary Fig. S1). The Harbour porpoises, absent in July and September, were present in other months, and their distribution and migrations are known to be associated with prey availability, primarily including Atlantic herring^94,95^. The scarcity of herring in July and September suggests that porpoises likely followed their prey out of the fjord during these months. On the other hand, the March sampling period also detected eDNA from freshwater fish like Northern pike (*Esox Lucius*), European perch (*Perca fluviatilis*), ruffe (*Gymnocephalus cernua*), and ninespine stickleback (*Pungitius pungitius*), which could be related to the melting snow, increasing the water coming from overflowing rivers and lakes into the Oslo fjord, and bringing eDNA from species not found in a marine environment^96^. Other factors like heavy rainfall or tidal flow direction could also contribute to variations^96^.

Many species in the Oslo fjord did not exhibit a clear pattern of seasonal presence, with non-migratory species like the two-spotted goby (*Gobiusculus flavescens*), goldsinny wrasse (*Ctenolabrus rupestris*), corkwing wrasse (*Symphodus melops*), and black goby (*Gobius niger*) consistently represented at each sampling event^90–93^. The continued presence of these species aligns with their known behaviour of residing in small social groups in the littoral zone, emphasising the importance of shallow water habitats^90,91^. Furthermore, species like goldsinny wrasse, thrive in rocky and vegetated substrates, and suitable habitat is known from around our sampling site in Drøbak, marked by underwater breakwaters and associated structures^94–96^. Among the species displaying migration patterns into the Oslo fjord during summer, Atlantic mackerel emerged as the most abundant, reaching a peak in September. This aligns with the expected migrations of Atlantic mackerel, which moves along the Norwegian coast after spawning in the Iberian Peninsula in early spring, foraging in the North Sea and Norwegian Sea^97–99^. Similarly, the eDNA signals reflect the migratory tendencies of garfish, characterised by their movement to shallower waters in April and May, followed by a return to the open ocean in the autumn^100^. Notably, garfish is known to arrive earlier than mackerel, which is also reflected in our results. Among the three species of *Pomatoschistus*, only the painted goby (*Pomatoschistus pictus*) displayed a relatively clear pattern of seasonal occurrence in the Oslo fjord, with lower abundance in March and January. While seasonal migrations have not been extensively described in this species, its close relative, the sand goby (*Pomatoschistus minutus*), is known for seasonal vertical migration in the Oslo fjord, staying in the littoral zone for breeding and hatchling growth between April and December^101^. The potential similarity in seasonal vertical migration patterns between painted goby and sand goby prompts the need for additional studies to unravel the intricacies of its behaviour in response to environmental conditions in the Oslo fjord.

In conclusion, such as the necessity for multiple markers to maximise diversity recovery and the ongoing gaps in our understanding of eDNA ecology, this study underscores the cost-effectiveness of eDNA metabarcoding. The findings align with established patterns of diversity in the Oslo Fjord for numerous taxa. The success of seasonal monitoring using eDNA, particularly in a predominantly marine system with seasonal freshwater input, is evident. These results emphasise the method's suitability for conservation and monitoring studies. The study, while acknowledging its constraints, provides a valuable contribution by demonstrating that seasonal monitoring in fjord environments effectively captures the dynamics of species behaviour and responses. This information is crucial for comprehending the complexities of the Oslo Fjord's dynamic ecosystem, offering valuable insights for future research and management strategies.

### Supplementary Information

Supplementary Information.

## Data Availability

Raw sequencing data can be found here: https://zenodo.org/doi/10.5281/zenodo.10381280. Additional information can be found in the supplementary information section.
