# Supplementary material for: Harnessing eDNA metabarcoding to investigate fish community composition and its seasonal changes in the Oslo fjord
Source: Sci Rep. 2024 May 2;14:10154. doi: 10.1038/s41598-024-60762-8 (PMC11065990; doi:10.1038/s41598-024-60762-8)
Supplement: Supplementary file 1 — Supplementary Information. [file 41598_2024_60762_MOESM1_ESM.pdf]

Harnessing eDNA metabarcoding to investigate fish community composition and its seasonal changes in the Oslo fjord

Cintia Oliveira Carvalho[1,2], William Gromstad[1], Micah Dunthorn[1], Hans Erik Karlsen[3], Audun Schrøder-Nielsen[1], Jonathan Stuart Ready[2], Torbjørn Haugaasen[4], Grete Sørnes[3], Hugo de Boer[1], Quentin Mauvisseau[1*]

[1] Natural History Museum, University of Oslo, Norway

[2] Group for Integrated Biological Investigation, Center for Advanced Studies of Biodiversity, Federal University of Pará, Belém, Brazil

[3] Marine Research Station Drøbak, University of Oslo, Norway

[4] Norwegian University of Life Sciences (NMBU), Faculty of Environmental Sciences and Natural Resource Management, P.O. Box 5003 NMBU, Aas, 1432, Aas, Norway

**Cintia Oliveira Carvalho and William Gromstad are joined first authors and contributed equally

*Corresponding authors:

Quentin Mauvisseau

quentin.mauvisseau@nhm.uio.no

Supplementary Figure S1. Heatmaps for the abundance of each species present in both markers at the different sampling events.

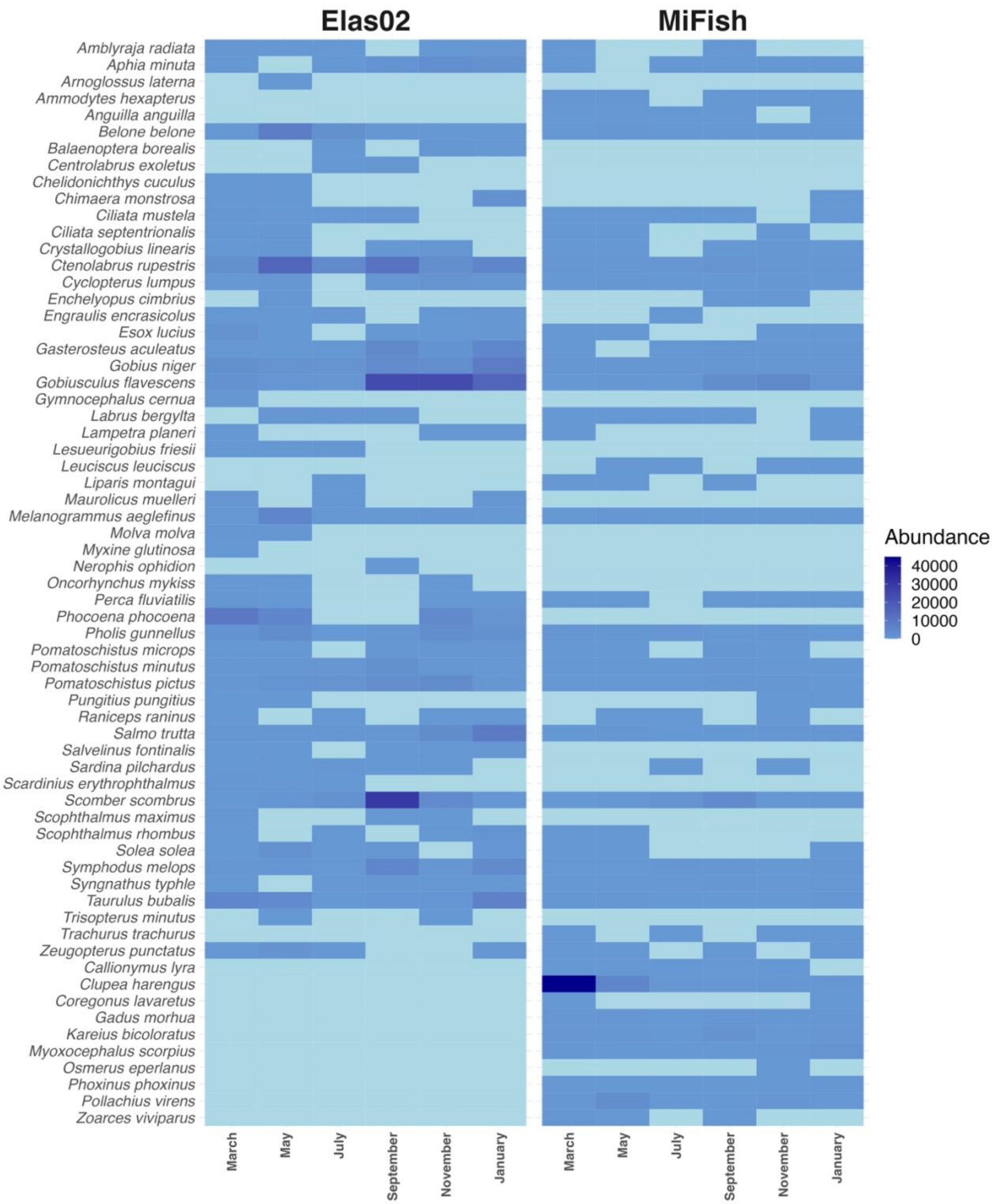

Supplementary Figure S2. Rarefaction and extrapolation sampling curves generated for Elas02 and MiFish datasets, considering both Field (A-B) and PCR replicates (C-D). The figures were constructed using 1000 bootstraps. Dashed lines on the curves indicate the extrapolated values of the diversity index obtained through extrapolation methods.

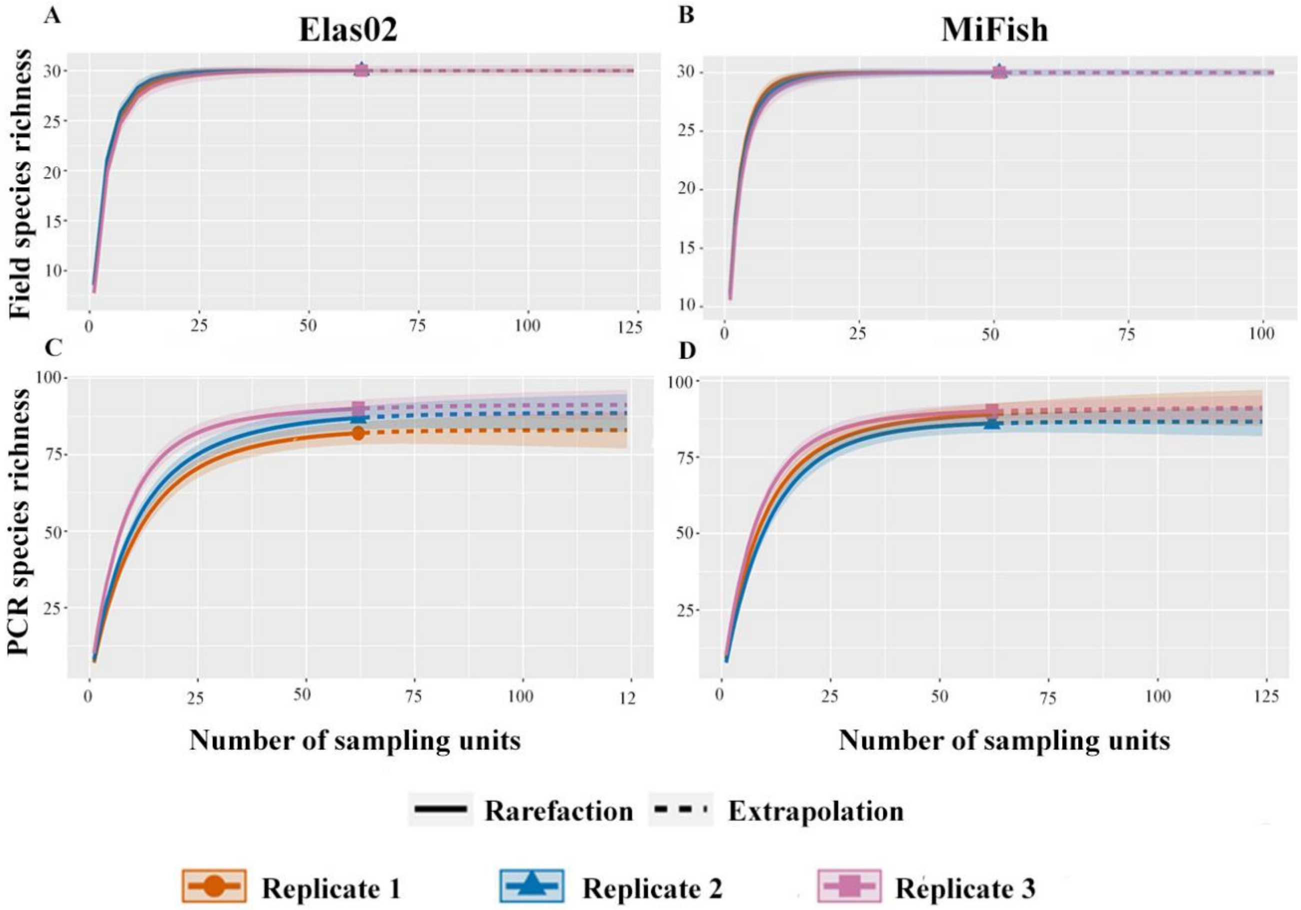

Supplementary Table S1. Overview of the sampled location, number of replicates collected, GPS coordinates, sampling dates and times, volumes of water filtered, water temperature, conductivity, salinity, Total Dissolved Solids, and pH.

Samples	Location	Replicate	Latitude	Longitude	Date	Time	Volume Filtered (mL)	Temperature	Conductivity (mS)	Salinity	TDS (g/L)	pH
1D1A1	A	1	59,66016	10,60527	08/03/2022	09:55	2000	3,6	24,03	25,6	26,66	6
1D1A2	A	2	59,66016	10,60527	08/03/2022	09:55	2000	3,6	24,03	25,6	26,66	6
1D1A3	A	3	59,66016	10,60527	08/03/2022	09:55	2000	3,6	24,03	25,6	26,66	6
1D1B1	B	1	59,66047	10,60952	08/03/2022	10:00	2000	3,9	25,1	26,4	27,3	6
1D1B2	B	2	59,66047	10,60952	08/03/2022	10:00	1500	3,9	25,1	26,4	27,3	6
1D1B3	B	3	59,66047	10,60952	08/03/2022	10:00	1500	3,9	25,1	26,4	27,3	6
1D1C1	C	1	59,66088	10,61419	08/03/2022	10:04	1500	3,5	23,62	25	26,06	6
1D1C2	C	2	59,66088	10,61419	08/03/2022	10:04	1400	3,5	23,62	25	26,06	6
1D1C3	C	3	59,66088	10,61419	08/03/2022	10:04	1500	3,5	23,62	25	26,06	6
1D1D1	D	1	59,66235	10,61814	08/03/2022	10:07	1400	3,3	23,28	24,7	25,87	6
1D1D2	D	2	59,66235	10,61814	08/03/2022	10:07	1500	3,3	23,28	24,7	25,87	6
1D1D3	D	3	59,66235	10,61814	08/03/2022	10:07	1500	3,3	23,28	24,7	25,87	6
1D1E1	E	1	59,66304	10,62322	08/03/2022	10:10	1500	3,3	23,02	25,3	26,4	6
1D1E2	E	2	59,66304	10,62322	08/03/2022	10:10	1500	3,3	23,02	25,3	26,4	6
1D1E3	E	3	59,66304	10,62322	08/03/2022	10:10	2000	3,3	23,02	25,3	26,4	6
2D1A1	A	1	59,66115	10,60482	11/05/2022	09:58	1400	10,9	25,69	21,2	21,93	7
2D1A2	A	2	59,66115	10,60482	11/05/2022	09:58	1400	10,9	25,69	21,2	21,93	7
2D1A3	A	3	59,66115	10,60482	11/05/2022	09:58	1400	10,9	25,69	21,2	21,93	7
2D1B1	B	1	59,66131	10,60948	11/05/2022	10:00	1200	10,9	25,14	21	21,77	7
2D1B2	B	2	59,66131	10,60948	11/05/2022	10:00	1100	10,9	25,14	21	21,77	7
2D1B3	B	3	59,66131	10,60948	11/05/2022	10:00	1200	10,9	25,14	21	21,77	7
2D1C1	C	1	59,66146	10,61477	11/05/2022	10:04	1400	10,9	25,18	20,9	21,72	7
2D1C2	C	2	59,66146	10,61477	11/05/2022	10:04	1400	10,9	25,18	20,9	21,72	7
2D1C3	C	3	59,66146	10,61477	11/05/2022	10:04	1400	10,9	25,18	20,9	21,72	7
2D1D1	D	1	59,66197	10,61994	11/05/2022	10:08	1400	10,9	25,02	20,8	21,63	7

2D1D2	D	2	59,66197	10,61994	11/05/2022	10:08	1400	10,9	25,02	20,8	21,63	7
2D1D3	D	3	59,66197	10,61994	11/05/2022	10:08	1400	10,9	25,02	20,8	21,63	7
2D1E1	E	1	59,66323	10,62332	11/05/2022	10:10	1400	10,9	25	20,8	21,57	7
2D1E2	E	2	59,66323	10,62332	11/05/2022	10:10	1400	10,9	25	20,8	21,57	7
2D1E3	E	3	59,66323	10,62332	11/05/2022	10:10	1400	10,9	25	20,8	21,57	7
3D1A1	A	1	59,66036	10,60519	04/07/2022	09:17	1200	19,3	30,54	21,6	22,29	7
3D1A2	A	2	59,66036	10,60519	04/07/2022	09:17	1200	19,3	30,54	21,6	22,29	7
3D1A3	A	3	59,66036	10,60519	04/07/2022	09:17	1200	19,3	30,54	21,6	22,29	7
3D1B1	B	1	59,66069	10,61004	04/07/2022	09:21	1200	19,1	30,52	21,6	22,33	6
3D1B2	B	2	59,66069	10,61004	04/07/2022	09:21	1200	19,1	30,52	21,6	22,33	6
3D1B3	B	3	59,66069	10,61004	04/07/2022	09:21	1300	19,1	30,52	21,6	22,33	6
3D1C1	C	1	59,66104	10,61441	04/07/2022	09:25	1200	19,1	30,42	21,5	22,2	6
3D1C2	C	2	59,66104	10,61441	04/07/2022	09:25	1200	19,1	30,42	21,5	22,2	6
3D1C3	C	3	59,66104	10,61441	04/07/2022	09:25	1200	19,1	30,42	21,5	22,2	6
3D1D1	D	1	59,66273	10,61811	04/07/2022	09:29	1200	19,3	30,43	21,5	22,18	7
3D1D2	D	2	59,66273	10,61811	04/07/2022	09:29	1200	19,3	30,43	21,5	22,18	7
3D1D3	D	3	59,66273	10,61811	04/07/2022	09:29	1200	19,3	30,43	21,5	22,18	7
3D1E1	E	1	59,66338	10,62304	04/07/2022	09:34	1200	19,4	30,4	21,4	22,13	7
3D1E2	E	2	59,66338	10,62304	04/07/2022	09:34	1200	19,4	30,4	21,4	22,13	7
3D1E3	E	3	59,66338	10,62304	04/07/2022	09:34	1200	19,4	30,4	21,4	22,13	7
4D1A1	A	1	59,66036	10,60519	09/09/2022	09:28	1000	17	30,49	22,8	23,48	6
4D1A2	A	2	59,66036	10,60519	09/09/2022	09:28	1000	17	30,49	22,8	23,48	6
4D1A3	A	3	59,66036	10,60519	09/09/2022	09:28	1000	17	30,49	22,8	23,48	6
4D1B1	B	1	59,66069	10,61004	09/09/2022	09:33	1000	16,9	30,72	23	23,6	6
4D1B2	B	2	59,66069	10,61004	09/09/2022	09:33	1000	16,9	30,72	23	23,6	6
4D1B3	B	3	59,66069	10,61004	09/09/2022	09:33	1000	16,9	30,72	23	23,6	6
4D1C1	C	1	59,66104	10,61441	09/09/2022	09:38	1000	16,8	30,34	22,8	23,37	6
4D1C2	C	2	59,66104	10,61441	09/09/2022	09:38	1000	16,8	30,34	22,8	23,37	6
4D1C3	C	3	59,66104	10,61441	09/09/2022	09:38	1000	16,8	30,34	22,8	23,37	6
4D1D1	D	1	59,66273	10,61811	09/09/2022	09:41	1000	17,1	30,97	23,1	23,72	6

4D1D2	D	2	59,66273	10,61811	09/09/2022	09:41	1000	17,1	30,97	23,1	23,72	6
4D1D3	D	3	59,66273	10,61811	09/09/2022	09:41	1000	17,1	30,97	23,1	23,72	6
4D1E1	E	1	59,66338	10,62304	09/09/2022	09:45	1000	17	31,27	23,5	24,05	6
4D1E2	E	2	59,66338	10,62304	09/09/2022	09:45	1000	17	31,27	23,5	24,05	6
4D1E3	E	3	59,66338	10,62304	09/09/2022	09:45	1000	17	31,27	23,5	24,05	6
5D1A1	A	1	59,66036	10,60519	17/11/2022	09:57	1400	8,8	24,38	22,2	22,99	7
5D1A2	A	2	59,66036	10,60519	17/11/2022	09:57	1400	8,8	24,38	22,2	22,99	7
5D1A3	A	3	59,66036	10,60519	17/11/2022	09:57	1400	8,8	24,38	22,2	22,99	7
5D1B1	B	1	59,66069	10,61004	17/11/2022	10:01	1400	8,8	24,33	22,1	22,97	7
5D1B2	B	2	59,66069	10,61004	17/11/2022	10:01	1400	8,8	24,33	22,1	22,97	7
5D1B3	B	3	59,66069	10,61004	17/11/2022	10:01	1400	8,8	24,33	22,1	22,97	7
5D1C1	C	1	59,66104	10,61441	17/11/2022	10:06	1400	9	25,02	22,6	23,39	7
5D1C2	C	2	59,66104	10,61441	17/11/2022	10:06	1400	9	25,02	22,6	23,39	7
5D1C3	C	3	59,66104	10,61441	17/11/2022	10:06	1400	9	25,02	22,6	23,39	7
5D1D1	D	1	59,66273	10,61811	17/11/2022	10:10	1400	9	25,22	23	23,77	7
5D1D2	D	2	59,66273	10,61811	17/11/2022	10:10	1400	9	25,22	23	23,77	7
5D1D3	D	3	59,66273	10,61811	17/11/2022	10:10	1400	9	25,22	23	23,77	7
5D1E1	E	1	59,66338	10,62304	17/11/2022	10:13	1400	8,8	24,67	22,4	23,2	7
5D1E2	E	2	59,66338	10,62304	17/11/2022	10:13	1400	8,8	24,67	22,4	23,2	7
5D1E3	E	3	59,66338	10,62304	17/11/2022	10:13	1400	8,8	24,67	22,4	23,2	7
6D1A1	A	1	59,66036	10,60519	18/01/2023	09:34	1300	3,2	22,94	24,5	25,68	7
6D1A2	A	2	59,66036	10,60519	18/01/2023	09:34	1300	3,2	22,94	24,5	25,68	7
6D1A3	A	3	59,66036	10,60519	18/01/2023	09:34	1300	3,2	22,94	24,5	25,68	7
6D1B1	B	1	59,66069	10,61004	18/01/2023	09:39	1300	2	21,27	23,5	24,85	7
6D1B2	B	2	59,66069	10,61004	18/01/2023	09:39	1300	2	21,27	23,5	24,85	7
6D1B3	B	3	59,66069	10,61004	18/01/2023	09:39	1300	2	21,27	23,5	24,85	7
6D1C1	C	1	59,66104	10,61441	18/01/2023	09:43	1300	2,2	21,34	23,4	24,71	7
6D1C2	C	2	59,66104	10,61441	18/01/2023	09:43	1300	2,2	21,34	23,4	24,71	7
6D1C3	C	3	59,66104	10,61441	18/01/2023	09:43	1300	2,2	21,34	23,4	24,71	7
6D1D1	D	1	59,66273	10,61811	18/01/2023	09:48	1300	2,1	21,25	23,2	24,52	7

6D1D2	D	2	59,66273	10,61811	18/01/2023	09:48	1300	2,1	21,25	23,2	24,52	7
6D1D3	D	3	59,66273	10,61811	18/01/2023	09:48	1300	2,1	21,25	23,2	24,52	7
6D1E1	E	1	59,66338	10,62304	18/01/2023	09:51	1300	2,3	21,67	23,6	24,9	7
6D1E2	E	2	59,66338	10,62304	18/01/2023	09:51	1300	2,3	21,67	23,6	24,9	7
6D1E3	E	3	59,66338	10,62304	18/01/2023	09:51	1300	2,3	21,67	23,6	24,9	7

Supplementary Table S2. Table with other species of potential interest detected by the Elas02 and MiFish primer sets during the metabarcoding analysis of the eDNA samples.

Species detected	**MiFish**	**Elas02**
Alca torda		X
Alces alces	X	X
Apodemus sylvaticus	X	X
Ardea cinerea		X
Arvicola amphibius	X	X
Balaenoptera borealis	X	X
Bos taurus	X	X
Canis lupus	X	X
Capra hircus	X	X
Capreolus capreolus	X	X
Castor fiber	X	X
Cervus elaphus	X	X
Coccothraustes coccothraustes	X	
Columba livia		X
Columba palumbus		X
Corvus corone	X	X
Cygnus olor	X	X
Dama dama		X
Equus caballus	X	X
Felis catus	X	X
Fringilla coelebs		X
Gallus gallus	X	X
Halichoerus grypus		X
Lepus timidus	X	
Luscinia svecica	X	X
Lutra lutra	X	
Melanitta americana		X
Melanitta deglandi	X	
Meleagris gallopavo	X	X
Mergus serrator		X
Microtus agrestis	X	X
Myodes glareolus	X	
Ovis aries	X	X
Parus major	X	
Phalacrocorax carbo	X	
Phocoena phocoena	X	X
Pyrrhula pyrrhula		X
Rangifer tarandus	X	X
Rattus norvegicus	X	X
Sciurus vulgaris	X	X
Scolopax rusticola		X
Somateria fischeri	X	
Sus scrofa		X
Uria aalge	X	X
Vulpes vulpes	X	X

Supplementary Table S3. Table with the species detected for each taxonomic assignment approach, SINTAX and Blast+, and by the Elas02 and MiFish primer sets during the metabarcoding analysis of the eDNA samples. In grey, the species only detected by Blast+.

				Elas02		Mifish	
**Order**	**Family**	**Gender**	**Species**	**SINTAX**	**Blast**	**SINTAX**	**Blast**
Anguilliformes	Anguillidae	*Anguilla*	*Anguilla anguilla*			X	
Artiodactyla	Balaenopteridae	*Balaenoptera*	*Balaenoptera borealis*	X			
Artiodactyla	Phocoenidae	*Phocoena*	*Phocoena phocoena*	X			
Beloniformes	Belonidae	*Belone*	*Belone belone*	X	X	X	X
Carangiformes	Carangidae	*Trachurus*	*Trachurus trachurus*		X	X	X
Chimaeriformes	Chimaeridae	*Chimaera*	*Chimaera monstrosa*	X	X	X	X
Clupeiformes	Clupeidae	*Clupea*	*Clupea harengus*		X	X	X
Clupeiformes	Clupeidae	*Clupea*	*Clupea pallasii*		X		
Clupeiformes	Clupeidae	*Sardina*	*Sardina pilchardus*	X	X	X	X
Clupeiformes	Engraulidae	*Engraulis*	*Engraulis encrasicolus*	X	X	X	X
Cypriniformes	Leuciscidae	*Leuciscus*	*Leuciscus leuciscus*		X	X	X
Cypriniformes	Leuciscidae	*Phoxinus*	*Phoxinus phoxinus*			X	X
Cypriniformes	Leuciscidae	*Scardinius*	*Scardinius erythrophthalmus*	X	X		
Esociformes	Esocidae	*Esox*	*Esox lucius*	X	X	X	X
Gadiformes	Gadidae	*Gadus*	*Gadus morhua*		X	X	X
Gadiformes	Gadidae	*Melanogrammus*	*Melanogrammus aeglefinus*	X	X	X	X
Gadiformes	Gadidae	*Pollachius*	*Pollachius virens*		X	X	X
Gadiformes	Gadidae	*Raniceps*	*Raniceps raninus*	X	X	X	X
Gadiformes	Gadidae	*Trisopterus*	*Trisopterus minutus*	X	X		
Gadiformes	Gaidropsaridae	*Enchelyopus*	*Enchelyopus cimbrius*	X	X	X	X
Gadiformes	Gasterosteidae	*Gasterosteus*	*Gasterosteus aculeatus*	X	X	X	X
Gadiformes	Gasterosteidae	*Pungitius*	*Pungitius pungitius*	X	X	X	X
Gadiformes	Lotidae	*Ciliata*	*Ciliata mustela*	X	X	X	X
Gadiformes	Lotidae	*Ciliata*	*Ciliata septentrionalis*	X	X	X	X
Gadiformes	Lotidae	*Molva*	*Molva molva*	X	X		
Gobiiformes	Gobiidae	*Aphia*	*Aphia minuta*	X	X	X	X
Gobiiformes	Gobiidae	*Crystallogobius*	*Crystallogobius linearis*	X	X	X	X
Gobiiformes	Gobiidae	*Gobius*	*Gobius niger*	X	X	X	X
Gobiiformes	Gobiidae	*Gobiusculus*	*Gobiusculus flavescens*	X	X	X	X
Gobiiformes	Gobiidae	*Pomatoschistus*	*Pomatoschistus microps*	X	X	X	X
Gobiiformes	Gobiidae	*Pomatoschistus*	*Pomatoschistus minutus*	X	X	X	X
Gobiiformes	Gobiidae	*Pomatoschistus*	*Pomatoschistus pictus*	X	X	X	X
Gobiiformes	Gobiidae	*Lesueurigobius*	*Lesueurigobius friesii*	X	X		

Labriformes	Labridae	*Ctenolabrus*	*Ctenolabrus rupestris*	X	X	X	X
Labriformes	Labridae	*Labrus*	*Labrus bergylta*	X	X	X	X
Labriformes	Labridae	*Symphodus*	*Symphodus melops*	X	X	X	X
Labriformes	Labridae	*Centrolabrus*	*Centrolabrus exoletus*	X	X		
Myxiniformes	Myxinidae	*Myxine*	*Myxine glutinosa*	X			
Osmeriformes	Osmeridae	*Osmerus*	*Osmerus eperlanus*		X	X	X
Perciformes	Cottidae	*Myoxocephalus*	*Myoxocephalus scorpius*		X	X	X
Perciformes	Cottidae	*Taurulus*	*Taurulus bubalis*	X	X	X	X
Perciformes	Cyclopteridae	*Cyclopterus*	*Cyclopterus lumpus*	X	X	X	X
Perciformes	Liparidae	*Liparis*	*Liparis montagui*	X	X	X	X
Perciformes	Percidae	*Gymnocephalus*	*Gymnocephalus cernua*	X	X		
Perciformes	Percidae	*Perca*	*Perca fluviatilis*	X	X	X	X
Perciformes	Pholidae	*Pholis*	*Pholis gunnellus*	X	X	X	X
Perciformes	Stichaeidae	*Chirolophis*	*Chirolophis ascanii*		X		
Perciformes	Triglidae	*Chelidonichthys*	*Chelidonichthys cuculus*	X			
Perciformes	Zoarcidae	*Zoarces*	*Zoarces viviparus*		X	X	X
Petromyzontiformes	Petromyzontidae	*Lampetra*	*Lampetra planeri*	X		X	
Pleuronectiformes	Bothidae	*Arnoglossus*	*Arnoglossus laterna*	X	X		
Pleuronectiformes	Pleuronectidae	*Kareius*	*Kareius bicoloratus*			X	
Pleuronectiformes	Scophthalmidae	*Scophthalmus*	*Scophthalmus maximus*	X	X		
Pleuronectiformes	Scophthalmidae	*Scophthalmus*	*Scophthalmus rhombus*	X	X	X	X
Pleuronectiformes	Scophthalmidae	*Zeugopterus*	*Zeugopterus punctatus*	X	X	X	X
Pleuronectiformes	Soleidae	*Solea*	*Solea solea*	X	X	X	X
Rajiformes	Rajidae	*Amblyraja*	*Amblyraja radiata*	X	X	X	X
Salmoniformes	Salmonidae	*Coregonus*	*Coregonus lavaretus*			X	
Salmoniformes	Salmonidae	*Oncorhynchus*	*Oncorhynchus mykiss*	X	X		
Salmoniformes	Salmonidae	*Salmo*	*Salmo trutta*	X	X	X	X
Salmoniformes	Salmonidae	*Salvelinus*	*Salvelinus fontinalis*	X	X		
Scombriformes	Scombridae	*Scomber*	*Scomber scombrus*	X	X	X	X
Stomiiformes	Sternoptychidae	*Maurolicus*	*Maurolicus muelleri*	X	X		
Syngnathiformes	Callionymidae	*Callionymus*	*Callionymus lyra*			X	X
Syngnathiformes	Syngnathidae	*Syngnathus*	*Syngnathus typhle*	X	X	X	X
Syngnathiformes	Syngnathidae	*Nerophis*	*Nerophis ophidion*	X	X		
Uranoscopiformes	Ammodytidae	*Ammodytes*	*Ammodytes hexapterus*			X	
Uranoscopiformes	Ammodytidae	*Hyperoplus*	*Hyperoplus lanceolatus*		X		X

Supplementary Table S4. Number of eDNA reads, combined for Elas02 and MiFish dataset, found for the six species showing seasonal presence in the Oslo fjord.

**Species**	**March**	**May**	**July**	**September**	**November**	**January**
Scomber scombrus	18	1323	1960	29697	3837	818
Pomatoschistus pictus	200	1566	1282	3087	3783	278
Phocoena phocoena	9982	5202	0	0	4119	1084
Maurolicus muelleri	398	0	1	0	0	668
Clupea harengus	44871	5756	181	169	698	270
Belone belone	11	9213	2468	220	4	4
